# Popular Health Almanac

**Published:** 1875-08

**Authors:** 


A "Popular Health Almanac."—As a counterblast to the innumerable quack medicine almanacs, the American Pharmaceutical Association propose a Popular Health Almanac.

The main aim of the undertaking is to counteract the demand for the thousands of vile nostrums, by giving to the consumer information on questions connected with public health, and making the pharmacist what he naturally should be, the medium of communicating this information for the benefit of the public, as well as for the advancement of his own business interests. The project can hardly fail to enlist the sympathy and support of all pharmacists who are not manufacturers of nostrums; and the energy of Dr. Hoffman, of the Chicago *Pharmacist*, if he will consent to act as editor of the Almanac for a number of years, will give and preserve for it a high character and lasting usefulness.

The recommendation of a series of prescriptions, adapted to popular use, to be placed in the hands of heads of families, was long urged in the *Reporter* as the true means of lessening the consumption of nostrums.—*Medical and Surgical Reporter.*

